# Evaluation of Microvascular Complications in Kidney Recipients With Posttransplant Diabetes Mellitus

**DOI:** 10.1210/clinem/dgad738

**Published:** 2023-12-15

**Authors:** Başak Çelik Kavaklılar, Özge Aybı Özdemir, Tolga Yıldırım, Özlem Dikmetaş, Hilal Toprak, Vedat Hekimsoy, Alperen Onur İşler, Rahmi Yılmaz, Sibel Kadayıfçılar, Yunus Erdem, Tomris Erbas, Uğur Ünlütürk

**Affiliations:** Department of Internal Medicine, Hacettepe University School of Medicine, Ankara 06100, Turkey; Department of Internal Medicine, Hacettepe University School of Medicine, Ankara 06100, Turkey; Department of Internal Medicine, Hacettepe University School of Medicine, Ankara 06100, Turkey; Department of Nephrology, Hacettepe University School of Medicine, Ankara 06100, Turkey; Department of Ophthalmology, Hacettepe University School of Medicine, Ankara 06100, Turkey; Department of Ophthalmology, Hacettepe University School of Medicine, Ankara 06100, Turkey; Department of Cardiology, Hacettepe University School of Medicine, Ankara 06100, Turkey; Department of Internal Medicine, Hacettepe University School of Medicine, Ankara 06100, Turkey; Department of Endocrinology and Metabolism, Hacettepe University School of Medicine, Ankara 06100, Turkey; Department of Internal Medicine, Hacettepe University School of Medicine, Ankara 06100, Turkey; Department of Nephrology, Hacettepe University School of Medicine, Ankara 06100, Turkey; Department of Ophthalmology, Hacettepe University School of Medicine, Ankara 06100, Turkey; Department of Internal Medicine, Hacettepe University School of Medicine, Ankara 06100, Turkey; Department of Nephrology, Hacettepe University School of Medicine, Ankara 06100, Turkey; Department of Internal Medicine, Hacettepe University School of Medicine, Ankara 06100, Turkey; Department of Endocrinology and Metabolism, Hacettepe University School of Medicine, Ankara 06100, Turkey; Department of Internal Medicine, Hacettepe University School of Medicine, Ankara 06100, Turkey; Department of Endocrinology and Metabolism, Hacettepe University School of Medicine, Ankara 06100, Turkey

**Keywords:** posttransplant diabetes mellitus, new onset diabetes after transplantation, NODAT, diabetic retinopathy, diabetic nephropathy, diabetic neuropathy, cardiac autonomic neuropathy, corneal confocal microscopy

## Abstract

**Context:**

The paucity of data on microvascular complications in patients with posttransplant diabetes (PTDM) is an obstacle to developing follow-up algorithms.

**Objective:**

To evaluate diabetic microvascular complications in patients with long-standing PTDM.

**Methods:**

In patients with ≥5-year history of PTDM and age-matched renal transplant recipients without PTDM (NDM), diabetic peripheral neuropathy was evaluated using the Michigan Neuropathy Screening Instrument, the CASE IV device, and in vivo corneal confocal microscopy (CCM). Cardiac autonomic neuropathy tests were performed using heart rate variability. Nephropathy screening was assessed using spot urine albumin/creatinine ratio and eGFR calculation. Diabetic retinopathy was evaluated by fundus examination and photography, and optical coherence tomography.

**Results:**

This study included 41 patients with PTDM and 45 NDM patients. The median follow-up was 107.5 months in the PTDM group. Peripheral neuropathy was significantly higher in the PTDM group than in the NDM group (*P* = .02). In the PTDM patients with peripheral neuropathy, corneal nerve fiber density examined by CCM was significantly lower than in PTDM patients without neuropathy (*P* = .001). Parasympathetic involvement was observed in 58.5% of the PTDM group and 22% of the NDM group (*P* = .001). Sympathetic involvement was present in 65.9% of the PTDM group and 29.3% of the NDM group (*P* = .001). Retinopathy was observed in 19.5% of patients in the PTDM group and in none of the NDM patients (*P* < .001). Renal functions were similar between the study groups.

**Conclusion:**

Cardiac autonomic neuropathy and diabetic retinopathy can affect patients with PTDM at a high rate. Diabetic retinopathy is a threat to the vision of PTDM patients. Diabetic peripheral neuropathy can be detected early in PTDM patients by CCM.

Diabetic retinopathy, nephropathy, and neuropathy are common complications of diabetes mellitus and share common mechanisms of development (1). Diabetic neuropathy is a broad-spectrum complication that includes autonomic neuropathy and distal symmetric peripheral neuropathy (2). Diabetic sensorimotor peripheral neuropathy can increase neuropathic pain, foot ulcers, lower extremity amputations, and mortality (3). The prevalence of diabetic sensorimotor peripheral neuropathy varies from 6% to 62% due to the adoption of different diagnostic criteria and differences in patient selection (4). Cardiac autonomic neuropathy is strongly associated with cardiovascular events, leading to morbidity and mortality in diabetic patients (5). The prevalence of cardiac autonomic neuropathy varies depending on different definitions, diagnostic tests, and evaluation of diverse population characteristics. It ranges from 17% to 66% in type 1 diabetes and from 31% to 73% in type 2 diabetes. Diabetic nephropathy is a major cause of end-stage renal disease and chronic kidney disease. It affects 40% of individuals with type 2 diabetes and 30% with type 1 diabetes (6). Diabetic retinopathy is a significant public health concern and a leading cause of blindness worldwide. The global prevalence of diabetic retinopathy is 22.2% (7).

Solid organ transplantation is becoming increasingly frequent as a result of advancements in immunosuppressive therapies and surgical procedures. While these advancements have resulted in an extended lifespan for patients, they have also created challenges in emerging complications that can affect the recipients in the long term. One common metabolic disorder that can develop after kidney transplantation is diabetes. Posttransplant diabetes (PTDM) can lead to microvascular and macrovascular events, reducing graft survival and increasing cardiovascular morbidity and mortality. Therefore, it is crucial to prevent, diagnose early, and adequately manage diabetes in these patients to avoid these complications (8).

It is highly recommended to screen for microvascular complications in both type 1 and type 2 diabetes patients to prevent or detect severe complications in the early period. However, the long development period of microvascular complications in diabetes, coupled with studies mainly focused on transplantation-related complications such as graft failure, has resulted in limited available data on PTDM patients (9). The limited data on microvascular complications and their development time in PTDM patients has resulted in a gap in the literature to develop follow-up algorithms.

Therefore, this prospective observational study aimed to evaluate diabetic microvascular complications in patients with long-standing PTDM using sensitive methods for each complication.

## Methods

This observational study prospectively enrolled patients under regular follow-up at the Departments of Endocrinology & Metabolism and Nephrology of Hacettepe University School of Medicine between 1985 and 2017. The inclusion criteria were being 18 years of age or older and not having a diagnosis of diabetes mellitus before transplantation. Patients diagnosed with PTDM and followed up for more than 5 years were included in the PTDM group. The diagnosis of PTDM was made by the American Diabetes Association guideline (10). A diagnosis of PTDM required fasting plasma glucose levels ≥126 mg/dL or glycated hemoglobin (HbA1c) ≥ 6.5%, or if 75 g oral glucose tolerance test was performed, a plasma glucose value ≥200 mg/dL at the second hour. In addition, patients must have received stable immunosuppressive treatment for at least 3 months following transplantation and have no active infection. Forty-one patients who agreed to participate in the study from 771 renal transplant recipients were included in the PTDM group. For the control group, 45 renal transplant recipient patients without a diagnosis of PTDM (the NDM group) from the pool of 771 renal transplant recipients were matched for age. The NDM group consisted of patients who had undergone kidney transplantation at least 5 years prior and who were confirmed to be nondiabetic based on a 75 g oral glucose tolerance test performed after an overnight 8-hour fast.

The ethical committee from the research board of Hacettepe University School of Medicine approved this study (GO 21/1309). All participants provided written, informed consent, and this study complies with the Declaration of Helsinki and the principles of Good Clinical Practice.

Patient interviews and electronic medical records were used to obtain demographic information such as age, gender, marital status, education level, occupation, smoking status, and family history of diabetes. Additionally, hospital records were reviewed to determine the causes of chronic kidney disease, comorbidities, pretransplant treatments, number of prior transplantations, donor type, presence and type of rejection, immunosuppressive treatments, any changes made, and the reasons for those changes.

The waist (cm) and hip (cm) measurements of the patients were taken, height (cm) and body weight (kg) were measured, and body mass index (BMI) was calculated using the formula body weight (in kilograms)/height^2^ (in meters). During the routine clinical follow-up of kidney transplantation, the patient's fasting plasma glucose (mg/dL), HbA1c (%), and kidney function tests, including creatinine and the glomerular filtration rate (GFR), were assessed. The GFR value was calculated using the CKD-EPI (Chronic Kidney Disease Epidemiology Collaboration) formula in mL/min/1.73 m². Additionally, the patient's total cholesterol (mg/dL), triglycerides (mg/dL), high-density lipoprotein (mg/dL), low-density lipoprotein (mg/dL), and thyroid-stimulating hormone (TSH) (uIU/mL) were measured.

The single point insulin sensitivity estimator (SPISE) index, which is used to estimate whole-body insulin sensitivity, was calculated following the formula (11):


$$\mathrm{SPISE}\;\mathrm{index}:\;(600\times \mathrm{HD}L^{0.185})/(\mathrm{Triglycerid}e^{0.2}\times \mathrm{BM}I^{1.338})$$

### Retinopathy Assessment

Eye fundus examination and fundus photography were used to evaluate the macula, optic nerve, retina, and retinal vascular structures for retinopathy. The findings were recorded and staged using the Zeiss 450 IR+ device with 7 fields, according to the 34-point scale. The Heidelberg Spectralis Optical Coherence Tomography device was used to detect diabetic macular edema and to examine the thinning of the retinal nerve fiber layer, which is a secondary finding of retinopathy (12). Central macular thickness and peripapillary retinal nerve fiber thickness were measured in microns. A trained ophthalmologist evaluated the images using the International Clinical Diabetic Retinopathy and Diabetic Macular Edema Disease Severity Scales, published in 2003, based on the Early Treatment Diabetic Retinopathy Study (13).

### Nephropathy Assessment

Two tests were conducted during nephropathy screening: eGFR calculation and urinary albumin to creatinine ratio (UACR) in spot urine. The eGFR was determined using the Chronic Kidney Disease Epidemiology Collaboration equation (14). For UACR, the following categories were used: <30 mg/g creatinine indicated no albuminuria, 30 to 300 mg/g creatinine showed microalbuminuria, and values >300 mg/g creatinine were considered overt albuminuria. If 2 out of 3 UACR specimens were abnormal within a 3- to 6-month period and/or if the eGFR was reduced, the patient was considered to have nephropathy.

### Neuropathy Assessment

The Michigan Neuropathy Screening Instrument (MNSI), a combined scoring system, was used to evaluate large and small fibers. This tool includes a 15-item self-administered MNSI questionnaire and a 4-item examination (MNSI clinical examination). Each foot is examined for deformities, dry skin/calluses, fissures, infections, ulcers, lack of vibratory sensation, and reflexes. Vibratory sensation was assessed using a 128-Hz tuning fork applied to the dorsal surface of the great toe. In the monofilament examination, correctly identifying 8 out of 10 applications of the 10-gram monofilament on each foot was considered normal. Patients who scored ≥4 on the questionnaire and ≥2.5 on the physical examination were considered to have diabetic peripheral neuropathy (15, 16).

Each participant in the study underwent a vibration test using the CASE IV device (WR Electronics, Minneapolis, MN, USA) to evaluate large fiber neuropathy by employing the 4-2-1 testing algorithm (17). The stimulator was placed on the dorsum of the left first toe proximal to the nail bed, and the stimulus range of the equipment was in 25 pre-programmed steps, ranging from 0 (no stimulus) to 25 (maximal stimulus). This method required a starting point of 13, an intensity standardized at the mid-strength of stimulus output by the equipment. If the patient perceived this stimulus, the stimulation strength was decreased by 4 steps. If still perceived, the stimulus was reduced by another 4 steps. If the stimulus was not perceived at the lower level, then the intensity was increased by 2 steps. If perceived, it was dropped by 1 step. In this way, a 4-2-1 testing algorithm was implemented. When the patient could barely distinguish between 2 levels separated by the lowest step of 1, that level was defined as the just noticeable difference (JND). The JND may also be reported in µm and ranked as a percentile.

The CASE IV device was also used to evaluate quantitative sensory tests to identify peripheral small fiber neuropathy. This device uses 25 different vibration levels and thermal stimulation, referred to as JND. The CASE IV system uses a set of 25 standardized vibration and thermal stimuli. During the test, the vibration and thermal probe were placed on the medial surface of the first metatarsophalangeal joint of the patient's right foot dorsum. The baseline level was set at JND-13. Before the test, patients were given a trial stimulus at the starting level (JND-13). Following the trial stimulus, 20 stimuli were presented, of which 5 were null stimuli (no stimulus given). The remaining stimuli increased by 1 JND, and a stepping algorithm was used to evaluate vibration and thermal detection thresholds. The test results were analyzed using normative data from the Rochester Diabetic Neuropathy Study of Healthy Subjects, which considered age, gender, anthropometric measurements, and measurement site. Patients with threshold values above the 99th percentile were classified as having abnormal threshold values (18).

In vivo corneal confocal microscopy (CCM) was performed on the right eyes of all patients. Before the examination, a sterile Viscotears gel (carbomer 0.2%) was applied to enable noncontact examination and eliminate optical interfaces. The Confoscan 4 microscope Z-link system (Nidek Technologies Padova, Italy) was used for the examinations by an experienced ophthalmologist. The CS4 software (Nerves Tracking Tool v1.3.0) was utilized for corneal fibril recognition and re-imaging. Corneal nerve fiber density (CNFD) in µm/frame and tortuosity coefficient (TC) were calculated using the Olivera-Soto and Efron scoring system (19). The TC was scored on a scale from 0 to 4 based on the degree of curvature. Grade 0 represented almost straight fibers, grade 1 described mildly curved fibers, grade 2 defined moderately dense and low-amplitude curved nerve fibers, grade 3 meant high-amplitude curvature, and grade 4 indicated tortuous fibrils.

All study participants underwent a 24-hour Holter using the ELA medical SpiderView (ELA Medical SORIN Group, Le Plessis-Robinson, France) Holter recorder with ELA medical flash card to record the ECG signals. The ELA medical SyneScope software was used for the noninvasive exploration of diabetic cardiac autonomic neuropathy, analyzing mean heart rate, time-domain, and frequency-domain heart rate variability (HRV) indexes according to European Society of Cardiology and the North American Society of Pacing and Electrophysiology (ESC/NASPE) guidelines. Non-sinus beats, electric noise, and artifacts were excluded from the analysis. The time-domain HRV parameters included the standard deviation of normal-to-normal intervals (SDNN), the standard deviation of the averages of normal-to-normal intervals in all 5 minutes (SDANN), the proportion of NN50 (number of successive normal-to-normal pair intervals that differ by more than 50 ms) divided by the total number of normal-to-normal intervals (PNN50), and the square root of the mean squared differences of successive normal-to-normal intervals (RMSSD), while frequency-domain HRV measures were low-frequency power and high-frequency power. Time-domain and frequency-domain HRV measures were compared to the normal values defined in the ESC/NASPE guidelines (20).

### Statistical Analysis

The normality of numerical variables was examined visually (histograms, probability plots) and analytically (Kolmogorov-Smirnov/Shapiro-Wilk tests). Descriptive analyses were used, such as mean ± SD for normally distributed numerical variables, median and interquartile range (IQR) for nonnormally distributed numerical variables, and frequency tables for ordinal variables. The Chi-square and Fisher Exact Chi-square tests were used to compare categorical data. Independent samples *t* test was applied for normally distributed variables between independent groups, while the Mann-Whitney U test was used for nonnormally distributed numerical variables. The correlation between numerical variables was investigated using Pearson and Spearman correlation analysis. The multivariate analysis used logistic regression analysis to examine the independent predictors. Diagnostic features of corneal confocal microscopy parameters were analyzed by receiver operating characteristics (ROC) curve analysis. A *P* value ≤ .05 was accepted as statistically significant. The statistical analysis was performed using Statistical Package for Social Sciences (SPSS) version 22 (IBM SPSS Inc., Chicago, IL).

## Results

**Figure 1.**
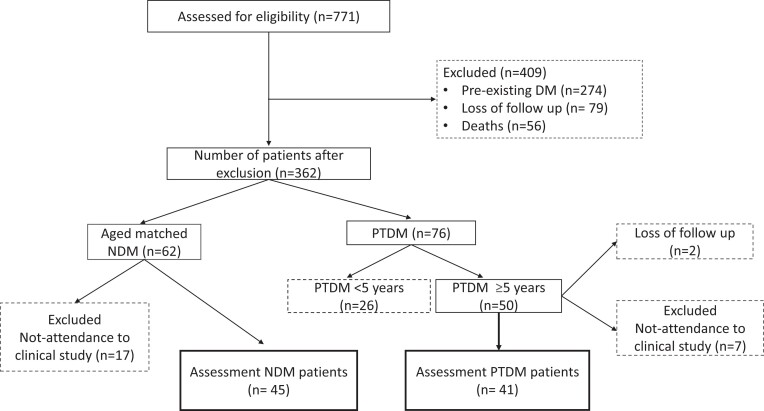
The flowchart of the inclusion process.

The data from 771 patients who underwent kidney transplantation between 1985 and 2017 were analyzed. Out of those, 41 patients with PTDM and 45 age-matched NDM controls were included in the study. The study flowchart is presented in Fig. 1. The demographic and clinical features of the patients are summarized in Table 1. The median follow-up duration from the transplantation dates was 107.5 months (IQR 92-156) for the PTDM group and 111.5 months (IQR 88-189) for the NDM group, indicating similar durations. Both study groups had similar mean ages and male-to-female ratios. The PTDM group had significantly higher BMI and waist circumference values than the controls. The etiology of chronic kidney disease was unknown in the highest proportion of cases in both groups (Table 1). In the PTDM group, the median age at diagnosis was 44.5 years (IQR 37-51.5), and the median duration of diabetes was 87.5 months (IQR 61-119). The median time to diagnosis after transplantation was 217 days (IQR 94-1207). Among the patients with PTDM, 1 patient (2.4%) did not receive any diabetes treatment. Medical nutritional therapy was received by 17% of patients, while 43.9% of patients were prescribed oral antidiabetic medication, and 59% required insulin therapy (Table 1).

**Table 1.** The demographic and clinical features of study groups

	PTDM (n = 41)	NDM (n = 45)	*P*
Female, n (%)	17 (41.5)	19 (42.2)	.94
Age, mean ± SD	48.9 ± 11.5	44.2 ± 11	.057
Body weight (kg), mean ± SD	79.3 ± 16.2	70.7 ± 13.3	.**008**
BMI (kg/m²), mean ± SD	29.8 ± 6.4	25.5 ± 5.1	**<**.**001**
WC (cm), mean ± SD	103.1 ± 17.3	92 ± 18.2	.**005**
Fasting PG (mg/dL), mean ± SD	118.6 ± 30.5	91.2 ± 11.9	**<**.**001**
HbA1c (%), mean ± SD	6.5 ± .8	5.5 ± .4	**<**.**001**
GFR (mL/min/1.73 m²), mean ± SD	50.8 ± 15.3	48.9 ± 16.6	.58
Creatinine (mg/dL), median (IQR)	1.16 (1.04-1.8)	1.20 (.9-1.7)	.49
T. cholesterol (mg/dL), mean ± SD	189.9 ± 46	204.6 ± 46	.75
LDL (mg/dL), mean ± SD	119.8 ± 29.8	132.2 ± 37.6	.09
HDL (mg/dL), mean ± SD	52.0 ± 13.3	48.9 ± 14.8	.30
Triglyceride (mg/dL), median (IQR)	133 (99-188)	157 (127-202)	.25
SPISE index, mean ± SD	5.3 ± 1.6	6.1 ± 1.7	.36
TSH (µIU/L), median (IQR)	1.60 (1-3)	1.60 (1.3-2.4)	.96
Hypertension, n (%)	25 (61)	34 (75)	.14
Systolic blood pressure, mmHg, median (IQR)	126 (120-140)	120 (110-135)	.**026**
Diastolic blood pressure, mmHg, median (IQR)	80 (71-85)	78.5 (70-82)	.76
Hyperlipidemia, n (%)	8 (19.5)	29 (64.4)	**<**.**001**
Coronary artery disease, n (%)	4 (9.8)	12 (26.7)	.**04**
Cerebrovascular disease, n (%)	1 (2.4)	3(6.7)	.61
Peripheral artery disease, n (%)	0	2 (4.4)	.49
Congestive heart failure, n (%)	0	2 (4.4)	.49
Metformin, n (%)	10 (24)	—	NA
SGLT-2 inhibitors, n (%)	2 (4.9)	—	NA
Insulin, n (%)	24 (44)	—	NA
Combination of insulin and metformin	6 (15)	—	NA
Diuretic, n (%)	9 (22)	5 (11.1)	.24
Beta blocker, n (%)	21 (51.2)	14 (31.1)	.06
Alpha blocker, n (%)	3 (7.3)	3 (6.7)	1
Calcium channel blocker, n (%)	11 (26.8)	10 (22.2)	.61
ACEI, n (%)	6 (14.6)	11 (24.4)	.25
ARB, n (%)	15 (36.6)	11 (24.4)	.22
Acetyl salicylic acid, n (%)	12 (29.3)	6 (13.3)	.07
Statin, n (%)	24 (58.5)	10 (22.2)	.**001**
Fibrate, n (%)	2 (4.9)	0	.22
**CKD etiologies, n (%)**			
Unknown	12 (29.3)	15 (33.3)	.68
Hypertension	5 (12.2)	4 (8.9)	.73
Nephritis	0	9 (20)	.**003**
Congenital	6 (14.6)	0	.**01**
Vesicourethral reflux	8 (19.5)	4 (8.9)	.15
Nephrolithiasis	3 (7.3)	3 (6.7)	1
Connective tissue	4 (9.8)	1 (2.2)	.18
FMF/Amyloidosis	0	3 (6.7)	.24
Polycystic kidney disease	1 (2.4)	2 (4.4)	1
Other	2 (4.9)	4 (8.9)	.67
**Renal replacement treatments, n (%)**			
Preemptive transplantation	10 (24.4)	16 (35.6)	.26
Hemodialysis	29 (70.7)	25 (55.6)	.18
Peritoneal dialysis	2 (4.9)	4 (8.9)	.67
**Donor types, n (%)**			
Cadaver	15 (36.6)	11 (24.4)	.22
Live	25 (61)	32 (71.1)	.32
Both of them	1 (2.4)	2 (4.4)	1
**Immunosuppressive therapy, n (%)**			
Prednisolone	39 (95.1)	43 (95.6)	1
Tacrolimus	13 (31.7)	34 (75.6)	**<**.**001**
Cyclosporine	19 (46.3)	7 (15.6)	.**002**
Sirolimus	3(7.3)	1 (2.2)	.34
Everolimus	5 (12.2)	7 (15.6)	.65
Mycophenolate mofetil	34 (82.9)	26 (57.8)	.**01**

Abbreviations: ACEİ, angiotensin converting enzyme inhibitors; ARB, angiotensin receptor blocker; BMI, body mass index; FMF, familial Mediterranean fever; GFR, glomerular filtration rate; HDL, high-density lipoprotein; LDL, low-density lipoprotein; NA, not applicable; NDM, patients without PTDM diagnosis; PG, plasma glucose; PTDM, patients with posttransplant diabetes mellitus; TSH, thyroid-stimulating hormone; WC, waist circumference.

Results from the MNSI questionnaire showed that the PTDM group had significantly higher mean scores than the NDM group (Table 2). The MNSI clinical examination evaluation also revealed that the mean score of the PTDM group was considerably higher than that of the NDM group (Table 2). The MNSI clinical examination assessment showed significantly more PTDM patients with peripheral neuropathy (scored 2.5 or higher) than NDM patients (Table 2). The 10-g monofilament test results indicated no significant difference between the study groups (Table 2). In the pinprick test, the painful responses differed significantly between the groups (Table 2). During the thermal and vibration quantitative sensory testing conducted using the CASE IV system, it was found that the mean of hot measurements was significantly lower in the PTDM group than in the NDM group. However, the 2 groups had no differences in cold measures (Table 2). In terms of thermal measurements, 4 patients (9.1%) in the PTDM group and 3 patients (7.7%) in the NDM group (*P* = 1.0) had results ≥99th percentile, which indicates the presence of small fiber neuropathy. In the vibration measurement, the median value for the PTDM group was lower than that of the NDM group (Table 2). Within the PTDM group, 4 patients (9.1%) had large fiber neuropathy, indicated by their ≥99th percentile. On the other hand, none of the patients within the NDM group had this condition. The results of the multifactorial regression analyses between microvascular complications events and various factors, including low-density lipoprotein, total cholesterol, triglyceride, HbA1c, and SPISE index, are presented in Table 3.

**Table 2.** Results of neuropathy evaluation in study groups

	PTDM (n = 41)	NDM (n = 45)	*P*
MNSI questionnaire, mean ± SD	.97 ± 1.1	.42 ± .72	.**008**
MNSI clinical examination, mean ± SD	1.14 ± 1.47	.28 ± .50	**<**.**001**
MSNI positive questionnaire, n (%)	2 (4.9%)	2 (4.4%)	1
MSNI positive clinical examination, n (%)	7 (17.1%)	1 (2.2%)	.**02**
**10 g monofilament, n (%)**			
Normal response	36 (87.8%)	38 (84.4%)	.43
Decreased response	4 (9.8%)	7 (15.6%)	
No response	1 (2.4%)	0	
**Pinprick test, n (%)**			
Painful	32 (78%)	44 (97.8%)	.**006**
Painless	9 (22%)	1 (2.2%)	
**Achilles reflex, n (%)**			
Normal	35 (85.4%)	42 (93.3%)	.29
Decreased	6 (14.6%)	3 (6.7%)	
**Toe muscle strength, n (%)**			
Normal	40 (97.6%)	45 (100%)	.47
Decreased	1 (2.4%)	0	
**Ankle muscle strength, n (%)**			
Normal	40 (97.6%)	45 (100%)	.47
Decreased	1 (2.4%)	0	
**Hot (JND), mean ± SD**	12.7 ± 6.2	15.7 ± 4	.**01**
**Cold (JND), mean ± SD**	7.5 ± 4.3	8.6 ± 4.7	.24
**Vibration (JND), median (IQR)**	7 (6-10)	12.6 (10.5-15)	**<**.**001**

Abbreviations: MSNI, Michigan Neuropathy Screening Instrument; NDM, Patients without PTDM diagnosis; PTDM, Patients with posttransplant diabetes mellitus.

**Table 3.** Multivariate analysis for predictors of microvascular complications events

	All participants			PTDM			NDM		
	***Diabetic Retinopathy***								
	**OR**	**95% CI**	***P* value**	**OR**	**95% CI**	***P* value**	**OR**	**95% CI**	***P* value**
LDL	0.96	0.91-1.01	.26	0.96	0.91-1.01	.26			
Total cholesterol	1.01	0.97-1.02	.58	1.02	0.97-1.00	.58			
Triglyceride	1.02	0.99-1.02	.28	1.01	0.99-1.02	.28			
HbA1c	3.29	1.20-8.79	.**01**	3.29	1.20-8.79	.**01**			
SPISE index	0.61	0.29-1.31	.21	0.61	0.29-1.31	.21			
	***Thinning of Retinal Nerve Fibers***								
LDL	0.98	0.93-1.12	.55	0.95	0.89-1.03	.25	1.02	0.91-1.11	.89
Total cholesterol	0.99	0.95-1.11	.78	1.04	0.97-1.06	.52	0.96	0.88-1.05	.45
Triglyceride	1.01	.99-1.02	.51	1.09	.98-1.01	.69	1.03	.98-1.01	.64
HbA1c	0.85	0.35-2	.73	1.48	0.46-4.70	.50	0.25	0.008-8.58	.44
SPISE index	0.56	0.31-1.01	.**05**	0.71	0.32-1.57	.40	0.35	0.12-0.97	.**04**
	***Diabetic Nephropathy***								
LDL	1.05	0.98-1	.39	1.02	0.96-1.05	.60	1.04	0.95-1.08	.59
Total cholesterol	0.99	0.97-1	.87	0.99	0.96-1.02	.73	1.02	0.95-1.05	.94
Triglyceride	1.01	0.99-1	.06	1.06	0.99-1.01	.31	1.12	0.99-1.04	.07
HbA1c	0.94	0.47-1.88	.86	0.90	0.36-2.22	.81	2.91	0.31-27.35	.35
SPISE index	1.4	0.96-2	.07	1.26	0.75-2.09	.37	1.71	0.88-3.30	.10
	***MSNI Positive Clinical Examination***								
LDL	1.02	0.95-1	.71	1.01	0.93-1.08	.87	1.02	0.90-1.17	.66
Total cholesterol	0.98	0.94-1	.52	0.98	0.93-1.04	.62	0.98	0.88-1.09	.76
Triglyceride	0.98	0.97-1	.13	0.98	0.97-1.00	.20	0.97	0.93-1.02	.28
HbA1c	1.33	0.48-3.71	.57	1.29	0.35-4.69	.69	0.82	0.008-84.26	.93
SPISE index	0.47	0.22-1.03	.06	0.40	0.13-1.19	.10	0.55	0.13-2.28	.41
	***Corneal Nerve Fiber Density*** *^a^*								
LDL	0.99	0.96-1.03	.86	1.01	0.95-1.07	.68	0.98	0.93-1.04	.69
Total cholesterol	1.01	0.99-1.04	.18	1.00	0.96-1.04	.96	1.02	0.98-1.07	.23
Triglyceride	0.99	0.98-1.00	.16	1.00	0.98-1.01	.79	0.99	0.98-1.00	.12
HbA1c	1.65	0.73-3.68	.22	1.17	0.38-3.63	.77	3.00	0.45-20.00	.25
SPISE index	0.88	0.62-1.24	.47	1.22	0.65-2.30	.52	0.71	0.44-1.15	.17
	***Cardiac Autonomic Neuropathy***								
LDL	0.97	0.94-1	.19	0.99	0.94-1.03	.65	0.96	0.90-1.03	.35
Total cholesterol	1.06	0.99-1	.24	1.06	0.97-1.03	.68	1.02	0.96-1.07	.48
Triglyceride	0.99	0.98-1	.21	0.99	0.98-1.00	.54	0.99	0.98-1.01	.53
HbA1c	1.84	0.90-3.74	.09	1.12	0.45-2.75	.79	3.02	0.24-37.57	.38
SPISE index	0.74	0.52-1	.11	0.84	0.51-1.36	.48	0.65	0.36-1.20	.17
	***Any Microvascular Complication***								
LDL	1.00	0.98-1	.28	1.07	0.96-1.05	.74	1.03	0.96-1.10	.38
Total cholesterol	0.99	0.96-1	.56	0.99	0.96-1.02	.68	0.99	0.94-1.05	.90
Triglyceride	1.02	0.99-1	.**03**	1.04	0.99-1.02	.14	1.01	0.99-1.03	.18
HbA1c	1.47	0.66-3.29	.34	1.42	0.50-4.04	.50	3.35	0.31-36.08	.31
SPISE index	1.32	0.89-1.98	.13	1.13	0.65-1.97	.64	1.57	0.79-3.10	.19

Abbreviations: HbA1c, glycated hemoglobin; LDL, low-density lipoprotein; MSNI, Michigan Neuropathy Screening Instrument; NDM, patients without PTDM diagnosis; PTDM, patients with posttransplant diabetes mellitus.

^*a*^A regression analysis was performed based on the cutoff value of corneal nerve fiber density (388.4 μm/mm^2^), which exhibited 78% sensitivity and 91% specificity in predicting diabetic neuropathy.

**Figure 2.**
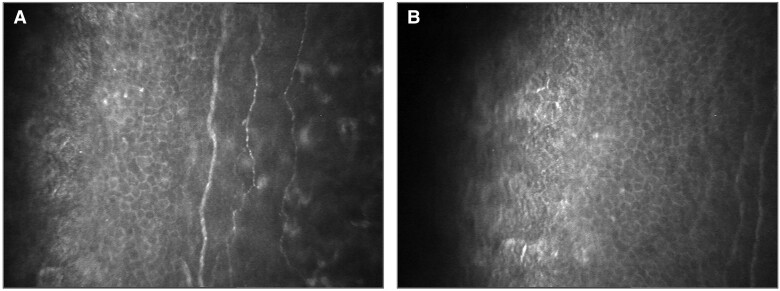
Images of subbasal nerves using in vivo confocal microscopy. A) Normal nerve plexus in a patient from the NDM group. B) Reduced plexus in a patient from the PTDM group.

**Figure 3.**
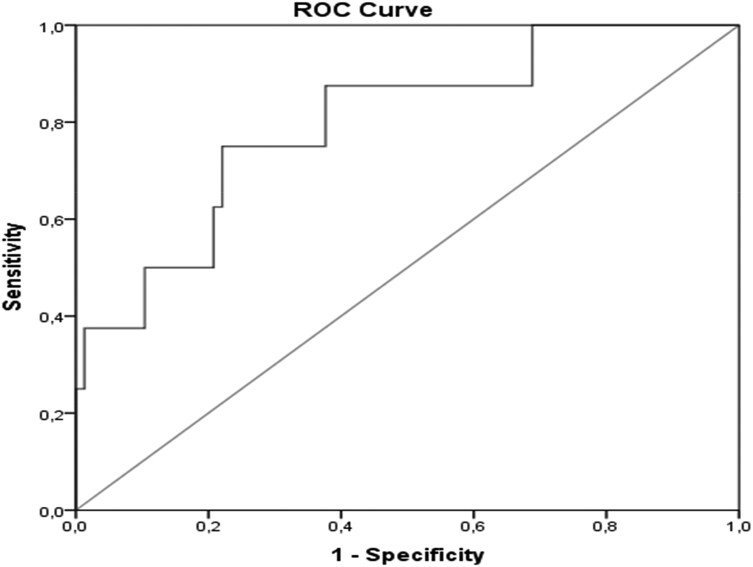
The receiver operating characteristic (ROC) curve of corneal nerve fiber density for predicting diabetic neuropathy (the area under the curve was .78, with a 95% CI of 0.55-1, *P* = .009).

When evaluating the corneal nerve fibers using CCM (Fig. 2), the mean CNFD and the median TC values were similar across all study groups (Table 4). However, when looking specifically at PTDM patients with neuropathy based on MNSI physical examination results, the CNFD was significantly lower than in those without neuropathy (Table 4). The tortuosity coefficient was also evaluated, but no significant difference was found between PTDM patients with and without neuropathy (Table 4). The accuracy of the diagnostic decision-making feature of CNFD in predicting diabetic neuropathy was evaluated using ROC curve analysis. The results showed that at a cutoff point of 388.4 µm/mm^2^, the sensitivity and specificity were 78% and 91%, respectively (Fig. 3).

**Table 4.** Results of in vivo corneal confocal microscopic evaluation in study groups

	PTDM (n = 41)	NDM (n = 45)	*P*
CFND µm/mm^2^, mean ± SD	560.0 ± 251.8	633.1 ± 461.0	.36
TC, median (IQR)	2 (1-3)	1 (1-2)	.54

	Patients with DN (n = 9)	Patients without DN (n = 32)	*P*
CNFD µm/mm^2^, mean ± SD	277.0 ± 213.0	620.1 ± 218.0	.**001**
TC, median (IQR)	2 (1-3)	1.5 (.5-3)	.37

Abbreviations: CFND, the mean corneal nerve fiber density; DN, diabetic neuropathy; NDM, patients without PTDM diagnosis; PTDM, patients with posttransplant diabetes mellitus; TC, tortuosity coefficient.

In the study using the ELA Medical SpiderView Holter device to assess cardiac autonomic neuropathy in patients, the heart rate was significantly higher in the PTDM group compared to the NDM group. Evaluating the parasympathetic system, it was found that the median PNN50 value was considerably lower in the PTDM group than in the NDM group, along with the median RMSSD value, which was also significantly lower in the PTDM group than in the NDM group. The median high-frequency measurement was also notably lower in the PTDM group compared to the NDM group (Table 5). On the other hand, the low-frequency measurement, which is used to evaluate the sympathetic system, was lower in the PTDM group compared to the NDM group. The SDNN and SDANN measurements were used to assess both the sympathetic and parasympathetic systems, and it was found that the mean value of SDNN was significantly lower in the PTDM group than in the NDM group. However, the median value of SDANN was higher in the PTDM group than in the NDM group. The 1996 ESC/NASPE guidelines for normal values were used to compare the involvement of the parasympathetic and sympathetic systems, and it was observed that the PTDM group had significantly higher involvement of both systems compared to the NDM group (Table 5).

**Table 5.** Cardiac autonomic neuropathy test results in study groups

	PTDM (n = 41)	NDM (n = 45)	*P*
HR (beats/min), mean ± SD	83.8 ± 15.0	76.8 ± 9.2	.**01**
PNN50 (%), median (IQR)	1.76 (.4-5.7)	8.96 (5.2-10.8)	**<**.**001**
RMSSD (ms) median, (IQR)	18.03 (13-28.5)	30.3 (24.6-36.7)	.**001**
SDNN (msn), mean ± SD	86.6 ± 38.4	103 ± 24.5	.**02**
SDANN (ms) median, (IQR)	96.6 (47-91)	89.4 (79-104)	.**003**
LF power (ms²) median (IQR)	313 (133-430)	642 (349-987)	**<**.**001**
HF power (ms²) median (IQR)	54 (24-111)	167 (93-481)	**<**.**001**
Cardiac parasympathetic autonomic dysfunction, n (%)	24 (58.5)	9 (20)	.**001**
Cardiac sympathetic autonomic dysfunction, n (%)	27 (65.9)	12 (26)	.**001**

Abbreviations: HF, high-frequency; HR, heart rate; LF, low-frequency; NDM, patients without PTDM diagnosis; PTDM, patients with posttransplant diabetes mellitus; PNN50, the proportion of nn50 divided by the total number of normal-to-normal intervals; RMSSD, square root of the mean squared differences of successive normal-to-normal intervals; SDNN, SD of normal-to-normal intervals; SDANN, SD of the averages of normal-to normal intervals in all 5 minutes.

The study groups had similar levels of microalbuminuria and overt albuminuria. No significant difference was found in the GFR of patients with microalbuminuria (Table 6).

**Table 6.** Posttransplant renal function and albuminuria in study groups

	PTDM (n = 41)	NDM (n = 45)	*P*
GFR (mL/min/1.73 m²), mean ± SD	50.8 ± 15.3	48.9 ± 16.6	.58
Creatinine (mg/dL), median (IQR)	1.16 (1.04-1.81)	1.20 (0.9-1.7)	.49
Microalbuminuria, n (%)	18 (43.9)	26 (57.8)	.19
Overt albuminuria, n (%)	5 (12.2)	7 (15.6)	.65
Spot urine alb/kre (mg/g), median (IQR)	104.8 (23-799)	84 (31-219)	.51

Abbreviations: GFR, glomerular filtration rate; NDM, patients without PTDM diagnosis; PTDM, patients with posttransplant diabetes mellitus.

In assessing retinopathy using fundus photography and optical coherence tomography, diabetic retinopathy was observed in 8 patients (19.5%), 4 of whom had proliferative retinopathy, in the PTDM group. In contrast, no retinopathy was detected in the NDM group (Table 7). Additionally, the evaluation of retinal nerve fibers using optical coherence tomography showed that the ratio of thinning of the retinal nerve fibers layer was similar between the groups (Table 7).

**Table 7.** Results of fundus photography and optical coherence tomography examinations in study groups

	PTDM (n = 41)	NDM (n = 45)	*P*
Retinopathy, n (%)	8 (19.5)	0	**<**.**001**
Thinning of retinal nerve fiber layer, n (%)	6 (14.6)	5 (11.1)	.62

Abbreviations: NDM, Patients without PTDM diagnosis; PTDM, Patients with posttransplant diabetes mellitus.

## Discussion

The present study evaluated the microvascular complications of PTDM in renal transplant recipients using sensitive methods for each microvascular complication. The results showed that the PTDM group had a significantly higher rate of neuropathy compared to the NDM group. Notably, the use of CCM to examine neuropathy in PTDM patients was reported for the first time in the literature. Although no significant difference was observed in the CNFD between the PTDM and NDM groups, a significant decrease in this parameter was detected in the PTDM patients who developed diabetic neuropathy. Furthermore, the PTDM group had a significantly higher incidence of both parasympathetic and sympathetic system involvement compared to the NDM group. Additionally, approximately 1 in 5 patients with PTDM had experienced retinopathy, with half of them having sight-threatening retinopathy, while no cases were observed in the NDM group.

In 2019, Londero et al conducted a study on a Brazilian cohort consisting of PTDM and NDM patients to assess microvascular complications. They found that the prevalence of diabetic neuropathy was higher in the PTDM group, using the MNSI clinical examination (21), which is similar to the findings of our study. However, their study reported a higher incidence of diabetic neuropathy in the PTDM group, which could be due to the higher average HbA1c levels (7% compared with 6.5% in our study). It is important to note that chronic kidney disease can induce neuropathy, but no further comments could be made since microvascular complications were not evaluated before transplantation in both studies.

Quantitative sensory tests are noninvasive diagnostic methods used to diagnose diabetic neuropathy. Although they are easy to apply, their accuracy can be affected by patient cooperation, algorithm variability, and susceptibility to anthropometric measurements. Unfortunately, many patients are only diagnosed in an advanced stage of neuropathy, limiting the potential benefits of early intervention (22). In vivo CCM is a noninvasive imaging technique that can evaluate the degeneration and regeneration processes of nerve fibers (23). In 2000, Rosenberg et al reported that individuals with type 1 diabetes had a decreased number of long nerve fiber bundles in the subbasal nerve plexus, which was related to the severity of diabetic peripheral neuropathy (23). The study highlights the potential of CCM as an early indicator of diabetic neuropathy. Although skin biopsy is commonly used to diagnose small fiber neuropathy, a comparison study showed that CCM evaluation is similarly sensitive (24). However, there is no agreement on which corneal nerve fiber parameter to use for diagnosing diabetic neuropathy and determining disease severity. Studies have indicated that CNFD is the most sensitive parameter in patients with type 1 and 2 diabetes (24-29).

Studies evaluating the use of CCM in assessing diabetic neuropathy in transplant recipients are limited to type 1 diabetic patients (30-32). These studies have shown improved CCM parameters after transplantation when diabetes is well-managed. However, the relationship between the tortuosity coefficient assessed by CCM and diabetic neuropathy is not yet clearly defined (33-35). The present study examined diabetic neuropathy using CCM in the PTDM patient group and found a significant decrease in CNFD in the PTDM patients who had developed neuropathy.

Cardiac autonomic neuropathy is a severe complication of diabetes that can significantly reduce the quality of life of patients and increase their morbidity and mortality rates (36). Our study found that the rate of cardiac autonomic neuropathy was higher in our cohort. The increased occurrence of cardiac autonomic neuropathy in the PTDM and NDM groups could be attributed to autonomic dysfunction caused by kidney insufficiency and transplantation (37). In Londero's study, no significant difference was found between PTDM patients and NDM groups in assessing cardiac autonomic neuropathy using the Ewing test (21). However, our study, which used a 24-hour Holter recording, showed a significant difference in cardiac autonomic neuropathy between the 2 patient groups. The Ewing test evaluates deep breathing and the Valsalva maneuver subjectively in relation to patient effort and can sometimes produce misleading results due to fluid retention causing hypotension (38). In contrast, a 24-hour HRV recording may be more sensitive in providing insights into circadian rhythms regulated by the sympathovagal system (39).

It has been reported that diabetic nephropathy affects around 40% of people with type 2 diabetes. A study conducted by Burrough et al examined 21 000 renal transplant recipients for complications over 12 years found that the rate of nephropathy was 31.3% (9). Similarly, Londero et al reported similar rates of nephropathy, and no significant differences were found between the PTDM and NDM groups (21). Our results were also compatible with these studies.

The prevalence rates of diabetic retinopathy vary greatly across different studies, ranging from 16% to 33%. Studies conducted in South America have reported the lowest observed prevalence of approximately 13% (7). According to the Wisconsin Epidemiologic Study of Diabetic Retinopathy, the prevalence of diabetic retinopathy was 25% at 5 years after diagnosis (40). In our study, almost 20% of patients in the PTDM group had retinopathy, while none of the NDM patients were affected. The duration of diabetes is closely related to the prevalence of diabetic retinopathy, and the average duration of diabetes was about 7 years in our study. Although the epidemiological studies included different types of diabetes, our study found an expected rate of diabetic retinopathy based on the average duration of diabetes.

According to a study conducted by Londero et al, none of the patients in the PTDM group had diabetic retinopathy (21). It is possible that this is due to the study being conducted in South America, where the incidence of diabetic retinopathy is the lowest. However, the study did report a significant thinning of the retinal fiber layer in the PTDM group (21). In contrast, our study observed thinning of the retinal fiber layer in both the PTDM and NDM groups. Additionally, it has been reported that retinal neuronal degeneration occurs in the early stages of diabetic retinopathy (21, 35).

The present study has a strong aspect, using specific and sensitive tests to evaluate each microvascular complication in PTDM patients. However, there are some limitations, like the relatively limited number of participants, the lack of a healthy control group, study groups not matching anthropometrically, and the inability to assess diabetic nephropathy histopathologically due to invasive procedures.

In conclusion, our study examined the development of microvascular complications in PTDM patients, where limited data exists in the literature. Cardiac autonomic neuropathy and diabetic retinopathy can affect patients with PTDM at a high rate after transplantation. Diabetic retinopathy was found to be a threat to the vision of PTDM patients. Diabetic neuropathy can be detected early in PTDM patients with CCM, a noninvasive and highly sensitive method. Considering the importance of early intervention in treating diabetic microvascular complications, our study contributed to the literature to close an essential gap in this field. Future research is needed to evaluate microvascular complications that cause morbidity and mortality in PTDM patients, and screening guidelines for microvascular complications specific to PTDM should be developed due to their unique characteristics compared with type 1 and type 2 diabetes.

## Data Availability

Some or all datasets generated during and/or analyzed during the current study are not publicly available but are available from the corresponding author on reasonable request.

## Abbreviations

BMI: body mass index
CCM: corneal confocal microscopy
CNFD: corneal nerve fiber density
eGFR: estimated glomerular filtration rate
ESC/NASPE: European Society of Cardiology and the North American Society of Pacing and Electrophysiology
GFR: glomerular filtration rate
HbA1c: glycated hemoglobin
HRV: heart rate variability
IQR: interquartile range
JND: just noticeable difference
MNSI: Michigan Neuropathy Screening Instrument
NDM: renal transplant recipients without posttransplant diabetes mellitus
PNN50: the proportion of NN50 divided by the total number of normal-to-normal intervals
PTDM: posttransplant diabetes mellitus
RMSSD: square root of the mean squared differences of successive normal-to-normal intervals
ROC: receiver operating characteristic
SDANN: standard deviation of the averages of normal-to-normal intervals
SDNN: standard deviation of normal-to-normal intervals
SPISE: single point insulin sensitivity estimator
TC: tortuosity coefficient
UACR: urinary albumin to creatinine ratio
